# Antithrombotic Therapy in Spinal Surgery Does Not Impact Patient Safety–A Single Center Cohort Study

**DOI:** 10.3389/fsurg.2021.791713

**Published:** 2022-01-26

**Authors:** Mohammed Banat, Johannes Wach, Abdallah Salemdawod, Gregor Bara, Ehab Shabo, Jasmin E. Scorzin, Martin Müller, Hartmut Vatter, Lars Eichhorn

**Affiliations:** ^1^Department of Neurosurgery, University Hospital Bonn, Bonn, Germany; ^2^Department of Emergency Medicine, Inselspital, Bern University Hospital, Bern University, Bern, Switzerland; ^3^Department of Anaesthesiology and Intensive Care Medicine, University Hospital Bonn, Bonn, Germany

**Keywords:** spinal surgery, spinal complications, antithrombotic therapy, patient safety, optimizing surgical planning

## Abstract

**Objective:**

Antithrombotic therapy is common in older patients to avoid thromboembolic events. Careful planning is required, particularly in the perioperative environment. There are no clearly date guidelines on the best timing for interrupting the use of anticoagulation in the case of spinal surgery. This study evaluates early per procedural clinical outcomes in patients whose antithrombotic therapy was interrupted for spinal surgery.

**Methods:**

This is a retrospective cohort study. All patients who underwent dorsal instrumentation from January 1, 2019 to December 31, 2020 were included. In group A, vitamin K antagonists (VKA) were suspended for 5 days and direct oral anticoagulants (DOAC) for 3 days. In group B, antiplatelet agents (APA) were paused for at least 7 days before surgery to prevent perioperative bleeding. Patients not taking anticoagulation medication were gathered into control group C. We analyzed demographic data, ASA status, blood loss, comorbidities, duration of surgery, blood transfusion, length of hospital stay, complications, thromboembolism, and 30 day in-hospital mortality. Multivariate analyses from the three groups were further analyzed and conducted.

**Results:**

A total of 217 patients were operated and included. Twenty-eight patients taking VKA/DOAC (group A), 37 patients using APA (group B), and 152 patients without anticoagulation (group C) underwent spinal surgery. Those using anticoagulants were significantly older and often with multimorbidity, but did not differ significantly in procedural bleeding, time of surgery, length of hospital stay, complication rate, thromboembolism, or 30 day in-hospital mortality (*p* > 0.05).

**Conclusion:**

Our data show that dorsal instrumentation safely took place in patients whose antithrombotic therapy was interrupted.

## Introduction

The perioperative management of patients receiving long-term anticoagulants such as vitamin K antagonists (VKA), oral factor IIa or Xa inhibitors (direct oral anticoagulation, DOAC), or antiplatelet agents (APA) is challenging (1, 2). The main reasons for the use of long-term anticoagulants are atrial fibrillation (AF), mechanical heart valves, or venous thromboembolisms. Atrial fibrillation is age-related, with an estimated prevalence of 1–4% in Europe, Australia, and the USA (3, 4).

Spinal dorsal instrumentation is an established treatment option for a range of spinal disorders, including trauma, neoplasia, adult degenerative diseases, and infection (5–7). Spinal surgery procedures have a high risk of major bleeding, and surgeons have to balance the risk of procedural bleeding against the increased thromboembolic risk if antithrombotic therapy is interrupted (8). Data on spinal surgery and VKA (e.g., warfarin, phenprocoumon), factor IIa-inhibitors (e.g., dabigatran), or factor Xa-inhibitors (e.g., apixaban, rivaroxaban, edoxaban) are limited. Antiplatelet therapy is controversial: some authors recommend discontinuation of APA before planned surgery (9–11), while others advocate its use (12). Currently, general recommendations are based on unquantified experience rather than on study results (13, 14).

This retrospective cohort study evaluates the per procedural risk of bleeding and of early thromboembolic events while antithrombotic therapy is interrupted during spinal surgery. Furthermore, operative time, need for blood transfusion, mean length of hospital stay, complication rates, thromboembolism, and 30 day in-hospital mortality are compared for each group.

## Methods

This retrospective clinical cohort study was approved by the university hospital's ethics committee (protocol no. 067/21). We analyzed patients who underwent complex dorsal instrumentation at our single center institution (Level 1 center for spinal surgery). We screened our database for patients who had complex dorsal instrumentation between January 1, 2019 and December 31, 2020 due to instable fracture, spondylodiscitis, tumor, or adult degenerative disease. We excluded patients with prosthetic heart valves, thromboembolism within the previous 3 months, arterial embolism within the previous month–because of the patient was not classified as operable at the time and risk of the operation was much too great, and those without a complete medication history.

As per the PAUSE study (**P**erioperative **A**nticoagulation **U**se for **S**urgery **E**valuation) (15) protocol, we did no preoperative coagulation testing and no heparin bridging as therapeutic anticoagulation, the patients had taken low dose heparin as bridging. Following in-house standards, APA were interrupted for 7–10 days and VKA (e.g., phenprocoumon) for at least 5 days. Direct oral anticoagulation (e.g., dabigatran, apixaban, rivaroxaban) was stopped 3 days prior to surgery. Similarly to a prospective multicenter study where 422 patients treated with required an invasive procedure (16), in our study clopidogrel was stopped 7 days before surgery and acetylsalicylic acid 10 days beforehand to avoid intra- and postoperative complications. There was no active preoperative compensation using prothrombin complex concentrate, fresh frozen plasma, or platelet concentrates. Patients only received blood transfusions during surgery if stipulated in standard patient blood management guidelines (17). The most of our patient collective had elective surgery, in cases of emergency operation with pathological coagulation; this was compensated before and not intraoperative.

We started the antithrombotic therapy directly on the first day after operation as low dose heparin as prophylaxis in the same procedure in all groups up to discharging. Patient mobilization was carried out directly after operation. Full therapeutic anticoagulation was routinely performed after discharging the patients, until then low dose heparin was given as prophylaxis.

Patients were retrospectively grouped according to their anticoagulants. Group A consisted of patients using oral anticoagulants (OAC) such as VKA or DOAC, and group B contained patients using APA. These patients were compared to those in control group C (patients without anticoagulants) to evaluate if anticoagulants and antithrombotic therapy impacted patient early periprocedural safety even when they were paused according to the usual guidelines.

Data on age, American Society of Anesthesiologists (ASA) physical status, Body Mass Index (BMI), blood loss, comorbidities, duration of surgery, blood transfusion, and length of hospital stay were extracted from patient records and operation reports. Data from the three groups were also analyzed for early surgical complications, need for surgical revision, thromboembolism, and 30 day in-hospital mortality in order to assess secondary short-term outcomes.

### Statistical Analysis

Statistical analyses were conducted using the computer software STATA® 16.1 (StataCorp, The College Station, Texas, USA). For descriptive analysis, the distribution of continuous variables was presented as the median accompanied by the interquartile range (IQR, 25th−75th percentile), as most variables were not normally distributed. Categorical variables were presented with the total number per category accompanied by the percentage. Associations between the different types of anticoagulation and categorical variables were tested with Chi-square tests. The Kruskal-Wallis test was used to compare the distribution of continuous variables between the three patient groups, with a pairwise Wilcoxon rank sum test as a *post-hoc* test. A *p* < 0.05 was considered significant.

Multivariable analysis of complications including confounders (i.e., age at surgery, Sex, BMI, ASA status, smoker, hypertension, diabetes, coronary artery disease, atrial fibrillation, pacemaker, creatinine pre-operative, preoperative Hb) was performed. Multivariable associations between type of anticoagulation and outcome (i.e., blood loss during surgery, length of hospital stay) were analyzed by models of linear regression (continuous outcomes). Relevant problems (i.e., postoperative internal problems such as thrombosis or sepsis, surgical revision needed within hospital stay) were analyzed using logistic regression (odds ratios as a measure of strength of association).

## Results

In total, 217 patients who were operated on between January 1, 2019 and December 31, 2020 were included in our study. Compared to group C, patients in group A (patients under VKA or DOAC; *n* = 28) were significantly older (*p* < 0.001), were more often smokers (*p* = 0.004) and male (*p* = 0.011), and suffered more frequently from coronary artery disease (CAD, *p* < 0.001) and atrial fibrillation (AF, *p* < 0.001). Those in group B (patients using APA; *n* = 37) were significantly older (*p* < 0.001), were more often smokers (*p* = 0.004) and more subject to hypertension (*p* < 0.001) and CAD (*p* = 0.004) than the patients in control group C (without anticoagulation; *n* = 152). There were no significant differences across the groups regarding smoking, diabetes mellitus, BMI, or diagnosis. Table 1 shows patient characteristics, comorbidities, and diagnoses.

**Table 1:** Univariable analysis of characteristics, comorbidities, and diagnoses.

		**Group A**	**Group B**	**Group C**	***p*** **-value**			
	**All (*n* = 217)**	**DOAC/VKA (*n* = 28)**	**APA (*n* = 37)**	**None (*n* = 152)**	**(A) vs. (C)**	**(A) vs. (B)**	**(B) vs. (C)**	**All**
Age at surgery [years]	69 (56–79)	79 (73–82)	76 (69–81)	61 (53–78)	<0.001	0.486	<0.001	<0.001
Gender, male	92 (42.2)	6 (21.4)	13 (35.1)	73 (47.7)	0.010	0.229	0.168	0.022
BMI [kg/m^2^]	26 (25–28)	26 (25–27)	27 (25–28)	26 (25–28)	0.451	0.097	0.137	0.205
ASA status	3 (2–3)	3 (3–4)	3 (3–3)	3 (2–3)	0.004	0.260	0.053	0.004
**Comorbidities**								
Smoker	81 (37.6)	4 (14.3)	12 (32.4)	66 (43.1)	0.004	0.093	0.235	0.012
Hypertension	94 (43.1)	15 (53.6)	22 (59.5)	57 (37.3)	0.105	0.635	0.014	0.024
Diabetes mellitus	41 (18.8)	4 (14.3)	11 (29.7)	26 (17.0)	0.723	0.143	0.079	0.166
Coronary artery disease	41 (18.8)	16 (57.1)	15 (40.5)	10 (6.5)	<0.001	0.184	<0.001	<0.001
Atrial fibrillation	39 (17.9)	21 (75.0)	12 (32.4)	6 (3.9)	<0.001	0.001	<0.001	<0.001
Pacemaker	11 (5.0)	7 (25.0)	2 (5.4)	2 (1.3)	<0.001	0.024	0.119	<0.001
**Diagnosis**								
Degenerative disease	80 (36.7)	10 (35.7)	18 (48.6)	52 (34.0)				
Malignancy	31 (14.2)	3 (10.7)	3 (8.1)	25 (16.3)				
Inflammation of vertebra and disc	42 (19.3)	9 (32.1)	7 (18.9)	26 (17.0)				
Trauma	65 (29.8)	6 (21.4)	9 (24.3)	50 (32.7)				

*Categorical variables are shown as number (%) and continuous variables as median (IQR)*.

*DOAC, Direct oral anticoagulation; APA, Antiplatelet agents; BMI, Body Mass Index; ASA, American Society of Anesthesiologists; IQR, Interquartile range (25th−75th percentile)*.

We found no significant differences in the amount of bleeding, time of surgery, or length of hospital stay between the groups. The amount of transfused blood products (packed red cells, fresh frozen plasma, or platelet concentrates) did not differ significantly across the groups (Table 2).

**Table 2:** Differences in surgical parameters.

		**Group A**	**Group B**	**Group C**	***p*** **-value**			
	**All (*n* = 217)**	**DOAC/VKA (*n* = 28)**	**APA (*n* = 37)**	**None (*n* = 152)**	**(A) vs. (C)**	**(A) vs. (B)**	**(B) vs. (C)**	**All**
Baseline Hb [g/dl]	12.1 (10.7–13.7)	11.5 (10.1–12.6)	12.6 (10.6–13.9)	12.3 (10.7–13.7)	0.094	0.257	0.981	0.259
Loss of blood during surgery [ml]	600 (300–1,000)	650 (300–1,100)	700 (300–1,200)	600 (300–1,000)	0.849	0.590	0.352	0.648
Intrahospital antibiotic therapy [% of all]	90 (41.3)	14 (50.0)	16 (43.2)	60 (39.2)	0.286	0.588	0.654	0.547
Blood transfusion [% of all]	76 (34.9)	12 (42.9)	14 (37.8)	50 (32.7)	0.297	0.683	0.551	0.534
Duration of surgery [min]	231 (170–300)	225 (171–308)	242 (170–286)	230 (170–300)	0.992	0.832	0.817	0.970
Length of hospital stay [days]	17 (10–26)	16 (12–30)	19 (10–26)	17 (10–25)	0.994	0.926	0.806	0.974

*Categorical variables are shown as number (%) and continuous variables as median (IQR)*.

*DOAC, Direct oral anticoagulation; APA, Antiplatelet agents; Hb, Hemoglobin; IQR, Interquartile range (25th−75th percentile)*.

The total complication rate was 30 out of 217 (13.82 %). Table 3 shows an overview of all complications recorded in our groups. In total, 14 early internal problems were recorded. Five patients developed an embolism (one deep vein thrombosis, four pulmonary embolisms) during their stay in hospital. Four of these patients were in group C and one was in group B. In group B (APA) there was one patient with early–directly postoperative cerebral stroke. Other ischemic events (i.e., myocardial infarcts or cerebral strokes) were not recorded. In total, 15 surgical complications were recorded.

**Table 3:** Complications after spinal surgery.

	**Complications**	**Anticoagulant therapy**		**No anticoagulation**	
		**DOAC/VKA (*n* = 28)**	**APA (*n* = 37)**	**None (*n* = 152)**	**Total (*n* = 217)**
Internal problems (*n* = 14)	Sepsis	0	2 (5.4)	1 (0.7)	3 (1.4)
	Pneumonia	0	0	2 (1.3)	2 (0.9)
	Heart failure	2 (7.1)	0	2 (1.3)	4 (1.8)
	Thromboembolism	0	1 (2.70)	4 (2.6)	5 (2.3)
Ischemic events (*n* = 1)	Cerebral stroke	0	1	0	1 (0.5)
Surgical problems (*n* = 15)	Cerebrospinal fistula	2 (7.1)	1 (2.70)	2 (1.3)	5 (2.3)
	Surgical revision needed	1	1		
	Postoperative bleeding	1 (3.6)	0 (0.0)	3 (2.0)	4 (1.8)
	Surgical revision needed	1		2	
	Wound infection	0 (0.0)	0 (0.0)	1 (0.7)	1 (0.5)
	Surgical revision needed			1	
	Disturbance of wound healing	2 (7.1)	2 (5.4)	1 (0.7)	5 (2.3)
	Surgical revision needed	2	1	1	
	No surgical revision	24 (85.7)	34 (91.9)	146 (96.1)	204 (94.0)
Total (*n* = 30)		28 (100.0)	37 (100.0)	152 (100.0)	217 (100.0)

No significant differences (*p* > 0.05) between DOAC/VKA therapy (group A) vs. no antithrombotic therapy (group C) or group B (APA) vs. no antithrombotic therapy (group C) could be found in multivariable analysis including several confounders (age, sex, BMI, ASA, Smoking status, preoperative hemoglobin, preoperative creatinine, hypertension, diabetes, coronary heart disease, and pacemaker). Antithrombotic therapy has no impact on blood loss during surgery, surgery duration, length of hospital stay, relevant problems (i.e., postoperative internal problems such as thrombosis or sepsis, surgical revision needed within hospital stay) using multivariable logistic, respectively, linear regression analysis as shown in Table 4.

**Table 4:** Multivariable analysis of complications including confounders (i.e., age at surgery, sex, BMI, ASA status, smoker, hypertension, diabetes, coronary artery disease, atrial fibrillation, pacemaker, creatinine pre-operative, preoperative Hb).

**Linear regression analysis**		**Coef**.	**Std. Err**.	**t**	**P>|t|**	**[95% Conf. Interval]**	
Blood loss during surgery	No anticoagulants	0	Base				
	Antiplatelet agent	258.8	142.8	1.8	0.07	−22.8	540.3
	DOAC/VKA	59.28	194.8	0.3	0.76	−324.8	443.4
Surgery duration	No anticoagulants	0	Base				
	Antiplatelet agent	11.8	22.4	0.5	0.60	−32.5	56.1
	DOAC/VKA	38.8	30.6	1.3	0.21	−21.6	99.1
Length of hospital stay	No anticoagulants	0	Base				
	Antiplatelet agent	4.3	3.5	1.2	0.22	−2.6	11.2
	DOAC/VKA	2.9	4.76	0.6	0.54	−6.5	12.3
**Logistic regression analysis**		**Odds Ratio**	**Std. Err**.	**Z**	**P>|z|**	**[95% Conf. Interval]**	
Any relevant complications (i.e., internal and/or surgical problems)	No anticoagulants	1	Base				
	Antiplatelet agent	3.3	2.0	1.9	0.05	0.99	11.1
	DOAC/VKA	2.7	2.2	1.2	0.24	0.52	13.4
Internal complications	No anticoagulants	1	Base				
	Antiplatelet agent	1.0	0.9	−0.02	0.99	0.15	6.2
	DOAC/VKA	0.5	0.6	−0.6	0.52	0.042	5.0
Surgical complications	No anticoagulants	1	Base				
	Antiplatelet agent	4.9	4.4	1.8	0.08	0.83	28.3
	DOAC/VKA	16.0	18.6	2.4	0.02	1.62	157.3

Six out of 217 patients died within 30 days of surgery. Neither DOAC/VKA (*n* = 2; *p* = 0.126) nor APA (*n* = 1, *p* = 0.483) were significantly associated with 30 day in-hospital mortality in univariable analysis. Causes of death were acute heart failure (*n* = 2), multiple organ failure (*n* = 2), and pneumonia and/or respiratory distress (*n* = 2).

In group C, we had pre-surgical 6 patients with comorbidities, 2 of them with pacer, and the other 4 of them with atrial fibrillation, nobody had anticoagulation, we did not find why, but we started after surgery in line with our internal algorithm the antithrombotic therapy.

Table 4 shows multivariate analysis of all complications recorded in our groups.

## Discussion

AF is one of the most common reasons for perioperative anticoagulation with VKA or DOAC. Interrupting anti-thrombic therapy for patients undergoing surgical procedures is challenging and surgeons have to assess the risk of procedural bleeding risk in order to decide whether to interrupt or continue anticoagulation. In this study VKA (e.g., phenprocoumon) was suspended >5 days prior to surgery, as recommended in previous studies (8, 18–20). Due to their shorter half-life and fewer interactions, DAOC (e.g., dabigatran, apixaban, rivaroxaban) were paused for 3 days before surgery (16). We also evaluated APA such as clopidogrel (suspended 7 days before surgery) and acetylsalicylic acid (stopped 10 days before surgery) (21). We questioned whether this interval would trigger increased blood loss or lead to unwanted thrombotic events.

### Vitamin K Antagonist (VKA) and Direct Oral Anticoagulation (DOAC)

Patients who interrupted their OAC therapy were significantly older and in a higher ASA classification than patients without anticoagulation. Nevertheless, we did not find any increase in risk of bleeding or of thromboembolic events periprocedural and in the time of hospital stay. Similarly, duration of surgery, length of hospital stays and 30-day mortality rate did not differ in the early short-term complication.

In general, the risk of procedural bleeding is dominated by the type of surgery, comorbidities, and medication (22–24). An individual's risk of bleeding can be quantified using scores such as HAS-BLED (25). A balance must be struck for each individual, between reducing the risk of thromboembolism and preventing bleeding (26, 27). Spinal surgery *per se* has a high bleeding risk (28) and discontinuation of VKA 4 days before and 4 days after surgery will produce about 8 days of sub-therapeutic anticoagulation (19). In the last decade newer oral anticoagulants such as dabigatran, rivaroxaban, and apixaban have been increasingly used. Interestingly, three large trials which compared DOAC (dabigatran, rivaroxaban, or apixaban) and warfarin for thromboembolic events in elective surgery found no clear differences. The thromboembolic risk during anticoagulant interruption was the same for dabigatran (29) and rivaroxaban as for warfarin (30). Only apixaban showed a lower rate of perioperative thromboembolism than warfarin (31). Nevertheless, more data are needed to guide physicians as to the optimal interruption intervals.

Although our cohort excluded patients with a high thromboembolic risk, it is worth mentioning that bridging (administration of a short-acting anticoagulant, typically a low molecular weight heparin, during interruption of a longer-acting agent) may in any case be necessary for patients using longer-acting anticoagulants. Interestingly, a meta-analysis of 12 cohort studies and six randomized trials found an increased risk of bleeding in patients who received bridging [relative risk (RR) 2.83; 95% CI 2.00–4.01] with no statistical reduction in thromboembolic risk (RR 1.26; 95% CI 0.61–2.58) (32). Preoperative bridging is therefore only recommended for individuals with a very high risk of thromboembolism (stroke within the past 3 months, mechanical heart valve, CHA2DS2-VASc score >6) (26). No patient in our cohort met these criteria.

### Antiplatelet Agents (APA)

For APA (i.e., platelet P2Y12 receptor blockers or aspirin) the decision to continue or to interrupt treatment also reflects an assessment of the balance between the risk of bleeding and the risk of ischemic events. The large randomized POISE-2 trial found an increased risk of bleeding but no improvement in cardiovascular or mortality outcomes when aspirin was continued perioperatively (33). Therefore, our center stopped clopidogrel 7 days before surgery and acetylsalicylic acid 10 days beforehand (21).

Although patients under APA were older, we did not see an increased risk of bleeding or of thromboembolic events, or any changes in the duration of surgery, length of hospital stay, or 30-day mortality rate. There were no significant differences in the amounts of transfused blood products (packed red cells, fresh frozen plasma, or platelet concentrates) we used.

### Complication Rates and Thromboembolism

The overall complication rate in our cohort was 13.82% (30 out of 217). The overall surgical complication rate was 6.9 %. Our values are in the low percentage range of what is found in the literature. A meta-analysis of patients with adult spinal deformities undergoing instrumentation and fusion found ranges between 4.9 and 29.6% for complications, especially dural tears (3–23.1%) and wound infection (6.5%) (34). Tram et al. reported an overall complication rate (including dural tears, transient neuralgia, hematoma at the epidural level, infection, and cardiovascular problems including embolism and thromboses) of about 0.47–6.9% (35).

We found no significant difference between the A, B, and C groups as regards the risk of thromboembolism. Our patients had a total thromboembolism risk of 2.3 % (5 out of 217). This is slightly higher than in the RE-LY study (1.2%) (33). However, Zhang et al. carried out a meta-analysis of 26 studies covering 3,216,187 patients following spinal surgery (36). They found a general incidence of thromboembolism in a range of 0.15–29.38 %. Factors such as increasing age, diabetes, chronic kidney disease, a preoperative non-ambulatory status, long duration of operation, spine fusion, and blood transfusion all increased the risk of venous thromboembolism. They did not find any association with BMI, hypertension, coronary heart disease, spondylolisthesis, intraoperative blood loss, type of surgical procedure (anterior lumbar interbody fusion, posterior intervertebral fusion, translaminar lumbar interbody fusion), or surgical site (36). Multivariable analysis showed a significant higher risk for surgical revisions needed in the VKA/DOAK group, but levels of significance was not reached in the APA group. Nevertheless, small statistical power and heterogenous reasons for surgical revision should be taken into account. In total we found no evidence for an increased thromboembolic risk when OAC or APA were interrupted.

## Limitation

The main limitations of this retrospective single center study were the small number of cases in each group. Thus, the study might not have had sufficient power to detect smaller differences between the anticoagulation groups. We looked at short-term outcomes and early complications, so our statement is limited to that time, certainly long-term studies are needed.

## Conclusion

Although spinal surgery has a high risk of intraprocedural bleeding, in our study acetylsalicylic acid interruption for 10 days, VKA and clopidogrel interruption for >5 days, and DOAC interruption for 3 days prior to surgery were effective at avoiding the periprocedural risk of serious bleeding, with no early negative effects on economy or patient safety. Furthermore, even in the absence of therapeutic bridging, there was no increased risk of thromboembolism in our short-term observation time.

## Data Availability Statement

The original contributions presented in the study are included in the article/supplementary material, further inquiries can be directed to the corresponding author.

## Ethics Statement

All procedures performed were in accordance with the Ethical Standards of the Institutional and National Research Committee (Ethic Committee of the Rheinische Friedrich Wilhelm's University Bonn) and with the 1964 Helsinki declaration and its later amendments or comparable Ethical Standards. Retrospective clinical cohort study. The investigation was approved by the Local Ethics Committee (protocol no. 067/21).

## Author Contributions

MB and LE: conceived, designed, performed the study, and data acquisition. MB: first drafting of the manuscript, illustrations, and wrote the manuscript. MB, LE, and MM: analysis and interpretation of data. JW, AS, GB, ES, JS, MM, LE, and HV: critical review of the manuscript. The final manuscript was critically revised and approved by all authors.

## Conflict of Interest

The authors declare that the research was conducted in the absence of any commercial or financial relationships that could be construed as a potential conflict of interest.

## Publisher's Note

All claims expressed in this article are solely those of the authors and do not necessarily represent those of their affiliated organizations, or those of the publisher, the editors and the reviewers. Any product that may be evaluated in this article, or claim that may be made by its manufacturer, is not guaranteed or endorsed by the publisher.

## Abbreviations

AF: Atrial fibrillation
APA: Antiplatelet agents
ASA: American Society of Anesthesiologists physical Status
BMI: Body Mass Index
CAD: Coronary artery disease
CT: Computer tomogram
DOAC: Direct oral anticoagulation
IQR: interquartile range (25th−75th percentile)
OAC: oral Anticoagulation
SD: Standard deviation
VKA: vitamin K antagonist.
